# Predicting Symptom Severities in Middle-Aged and Older Adults With Arthritis and Multimorbidity

**DOI:** 10.1093/geroni/igab046.705

**Published:** 2021-12-17

**Authors:** Wenhui Zhang

**Affiliations:** Emory University, Atlanta, Georgia, United States

## Abstract

Introduction: Uncertainties increase with disease guideline-driven decision-making for older adults as their numbers of chronic conditions and functional limitations increase. A national study found that people with arthritis plus ≥ one other chronic condition have reported significantly higher social participation restriction, serious psychological distress, and work limitation than those with ≥two non-arthritis chronic conditions. However, how arthritis comorbidities contribute to the symptoms such as pain, fatigue, sleep, depression, anxiety, and cognitive abilities that chronically impair people’s daily functioning remain unexplored. Purpose: To explore how arthritis comorbidities predict the symptom severities of pain interference, fatigue, sleep disturbance, depression, anxiety, and cognitive abilities among community-dwelling middle-aged and older adults. Method: 140 community people aged over 50 with arthritis and multimorbidity were recruited. Stepwise regressions predicted the PROMIS symptoms of pain interference, fatigue, sleep disturbance, depression, anxiety, and cognitive abilities with arthritis type and 18 comorbidities measured by the Functional Comorbidity Index checklist after controlling for demographics. Results: Obesity, chronic obstructive pulmonary disease, diabetes, and income significantly predicted pain interference (adjusted R2=35%). Marital status, obesity, and peripheral vascular disease significantly predicted fatigue (adjusted R2=17%). Depression diagnosis and income adequacy significantly predicted depressive symptoms (adjusted R2=23%). Depression, income adequacy, and anxiety diagnosis significantly predicted anxiety (adjusted R2=23%). Age significantly predicted cognitive abilities (adjusted R2=12%). Discussion: Comorbidities and socio-demographics, especially income, impact symptom experiences of people aging with arthritis and multimorbidity. Future studies should explore the pathogenesis among arthritis, comorbidities, and symptoms for tailored intervention while disclosing health disparities associated with the arthritis multimorbidity.

